# Starvation induces hepatopancreas atrophy in Chinese mitten crab (*Eriocheir sinensis*) by inhibiting angiogenesis

**DOI:** 10.1186/s12864-023-09620-x

**Published:** 2023-10-12

**Authors:** Hongli Liu, Yang Feng, Ma Yang, Ya Huang, Minghao Li, Yi Geng, Ping Ouyang, Defang Chen, Shiyong Yang, Lizi Yin, Liangyu Li, Xiaoli Huang

**Affiliations:** 1 Department of Aquaculture, College of Animal Science & Technology, Sichuan Agricultural University, Chengdu, Sichuan 611130 China; 2 Department of Basic Veterinary, College of Veterinary Medicine, Sichuan Agricultural University, Chendu, Sichuan 611130 China; 3 Fisheries Research Institute, Chengdu Academy of Agriculture and Forestry Sciences, Chengdu, Sichuan 611130 China

**Keywords:** Hepatopancreas, Starvation, *Eriocheir sinensis*, Angiogenesis

## Abstract

**Background:**

The hepatopancreas of crustaceans serves as a significant organ for both the synthesis and secretion of digestive enzymes, as well as energy storage. In the event of food shortage, the hepatopancreas can provide energy for survival. To investigate the potential regulatory mechanisms of the hepatopancreas in response to starvation in *Eriocheir Sinensis*, transcriptome analysis, histological study and qRT-PCR were performed.

**Results:**

The results showed that starvation caused a decrease in the hepatopancreas index of *E. sinensis*, which had certain effects on the tissue structure, metabolism and angiogenesis in the hepatopancreas. In addition, WGCNA and linear regression analysis showed that the genes significantly related to the hepatopancreas index were mainly enriched in the angiogenesis pathway, in which AKT signaling played an important role. Starvation may inhibit AKT signaling pathway by reducing the expression of TGFBI, HSP27, HHEX, and EsPVF1, thereby hindering angiogenesis, promoting apoptosis, and leading to hepatopancreas atrophy.

**Conclusion:**

These results indicate that AKT plays an important role in the angiogenesis pathway and apoptosis of the starvation induced hepatopancreas index reduction, which is beneficial to further understand the effect of starvation stress on hepatopancreas of Chinese mitten crab.

**Supplementary Information:**

The online version contains supplementary material available at 10.1186/s12864-023-09620-x.

## Introduction

The Chinese mitten crab (*Eriocheir sinensis*), a member of the family Varunidae, which grows mainly in the coastal estuaries from the Korean Peninsula to Fujian, China, is one of the most important freshwater economic crabs in China [1]. Due to its delicious meat and rich nutrition, it has been a valuable aquatic product loved by the Chinese people with annual output as high as 7.76 × 10^5^t, accounting for 18.22% of the crustacean economic animals (Fisheries statistical yearbook, 2021).

The breeding cycle of Chinese mitten crab is usually 2 years, with a 2–3 month overwintering period. However, it is difficult to estimate the breeding density of Chinese mitten crab under the intensive pond arrangement and paddy field integrated culture mode, which often leads to body starvation during overwinter due to insufficient food intake [2, 3]. At present, starvation has become one of the physiological pressures that crustaceans often suffer during their growth and development. Under starvation stress, the digestive enzyme activity, tissue structure and material composition of crustaceans will undergo adaptive changes [2]. Prolonged starvation tends to lead to reduced survival and molt rate of crustaceans [2]. Like most crustaceans, hepatopancreas are important lipid storage tissues in *Eriocheir sinensis* [3]. Starvation may have a similar effect on the hepatopancreas of shrimp and crabs. In a study of *Cherax quadricarinatus*, starvation was found to cause the stellate lumen of the hepatopancreas to enlarge while reducing the fat content in the R-cells [4]. In addition, the number of B-cells increased, the number of R-cells decreased, the stellate lumen enlarged, and a large number of eosinophilic inclusions were observed in the hepatopancreas of the crab after starvation (7d) [5]. However, no obvious changes in F-cells and other structures were found [5]. When the duration of starvation reaches 21 days, hepatopancreas fat content and hepatopancreas index of Chinese mitten crab significantly decreased, thus activating fatty acid metabolism pathway and active energy catabolism in vivo, and finally consuming hepatopancreas cells through apoptosis to maintain energy metabolism. [5]. Therefore, energy metabolism is the main pathway of hepatopancreas atrophy caused by starvation. However, whether other possible mechanisms other than energy metabolism affect the hepatopancreas of crabs still remain unknown.

In living organisms, it is usually necessary to synthesize biological macromolecules through glucose and lipid metabolism and consumption of glutamine to provide energy for growth and proliferation to maintain cell survival [6]. Without a source of nutrients, these cells quickly decompose themselves, leading to cell death. Previous studies had suggested that the circulatory system of the crab is “open”, but recent studies have found that the circulatory system of the crab should be defined as an “incomplete closed system” [7, 8]. Pairs of hepatic arteries branch within the hepatopancreas to form small arteries, which in turn split into tiny capillaries, and these small vessels may actually form a true capillary networks [7]. Under nutrient starvation conditions, upregulated histone demethylase JHDM1D can inhibit cell growth by suppressing angiogenesis, and thus lead to apoptosis [9, 10]. Inhibition of angiogenesis also occurs under dietary restriction (a condition that limits energy supply but does not result in nutrient deficiency) [11, 12]. This suggests that starvation may inhibit angiogenesis. Based on previous studies, chronic starvation has been found to cause atrophy in the hepatopancreas of *E. chinensis*, a process caused mainly through apoptosis involving genes related to the mitochondrial pathway (e.g., *Bax* and *AIF*) as well as genes related to the death receptor pathway (e.g., *caspase-8*) [5]. However, whether this apoptosis is exacerbated by angiogenic obstruction remains unknown. As a consequence, a thorough understanding of the regulatory mechanism of starvation stress on hepatopancreas in Chinese mitten crab is essential, which is conducive to providing therapeutic targets for the corresponding diseases.

## Materials and methods

### Animals

200 *E. sinensis*, with randomized sex and body weight of 13.35 ± 3.09 g, were procured from a farm in Jiangsu, China. One week before the experiment, all *E. sinensis* were separately transferred to culture boxes with sizes of 19.0 cm × 12.5 cm × 7.5 cm for rearing to adapt to the environment. During the feeding process, the *E. sinensis* are fed daily at 18:00, and the amount of feed is calculated based on 5% of the body weight of the *E. sinensis*. Furthermore, a daily renewal of 20% of the rearing water is implemented.

### Experiment design

At the end of environmental acclimatization, 50 *E. sinensis* with sound limbs and good vigor were selected for the experiment. The experiment was conducted as follows: the selected *E. sinensis* were randomly divided into control and starvation groups, where the starvation group was not fed with feed, while the control group was fed with commercial feed (Tongwei, China). According to the previous study, the hepatopancreas index of *E. sinensis* decreased significantly when it was starved for 21 days, and the corresponding experimental conditions were set accordingly [5]. The experimental period was 21 days, during which the temperature was maintained at 19 ± 3 °C, the pH was 7.5–7.8, the dissolved oxygen (DO) was 6.5-6.9 mg/L and the feeding method was the same as the environmental acclimation period. After the experiment, 12 *E. sinensis* were randomly selected from each group for detection, including RNA-seq detection, HE staining, qRT-PCR and hepatopancreas index (HIS) calculation, as described below. Hepatopancreas for RNA-seq and qRT-PCR were placed in liquid nitrogen for rapid freezing immediately after removal and stored at -80 °C until use. During the experiment, the *E. sinensis* were always in the intermolt period.

### H&E stain

After the experiment, the hepatopancreas of 12 *E. sinensis* in each group were collected and fixed in AFA Davidson fixative [13]. And the fixed hepatopancreas was trimmed into blocks, placed in embedding boxes, dehydrated in graded ethanol solution, xylene removed, paraffin embedded, and made into 4 μm sections, which were then stained with hematoxylin and eosin (Besso Biotechnology, Zhuhai, China).

### RNA extraction, library construction and sequencing

Three *E. sinensis* were randomly selected from each of the starvation and control groups, and the total RNA in their hepatopancreas was extracted according to the instructions of Trizol reagent (Invitrogen, USA), and the total RNA quality was detected by 1% agarose gel electrophoresis and Nanodrop 2000 (Thermerfly, USA). Illumina TruseqTM RNA sample prep Kit was used to construct the library. The construction of the library was carried out in accordance with the following specifications: a minimum quantity of 1 µg total RNA, a concentration of at least 35 ng/µL, and a minimum OD260/280 and OD260/230 ratios of 1.8 and 1.0, respectively. After the library was qualified, Illumina NobaSeq 6000 was used for high throughput sequencing, and the sequencing mode was PE150. The specific process of library construction and sequencing was completed by Shanghai Meiji Biological Company.

### RNA-seq data analysis

The raw data obtained by sequencing were quality controlled using fastp to obtain clean read segments. Then, an index of the *E. sinensis* reference genome was constructed using TopHat2, and the clean read segments were compared with the reference genome (http://www.genedatabase.cn/esi_genome.html) to obtain information on the positioning of the read segments on the reference genome. Next, transcript read counts were calculated for each sample using RSEM. Subsequently, the number of read counts in each sample was normalized and transcript expression levels were analyzed using the DESeq2 software package. In order to mitigate the incidence of false positives, screening criteria were employed, requiring statistical significance with a p-value of less than 0.05 and an absolute logarithmic fold change of greater than 2. In addition, the Gene Ontology (GO) and Kyoto Encyclopedia of Genes and Genomes (KEGG) databases were utilized for functional annotation of the screened differentially significant genes (*P < 0.05*) [14–17].

### Quantitative real-time PCR (qRT-PCR)

To verify the accuracy of the transcriptome sequencing results, five Chinese mitten crabs in each group were selected and validated using qRT-PCR. The operation was similar to the previous study [18]. Briefly, total RNA was extracted from the hepatopancreas of *E. sinensis* using the Animal Total RNA Extraction Kit (Foregene, Shanghai, China), and then reverse transcribed into cDNA using RT Easy™ II (with gDNase) (Foregene, Shanghai, China) according to the instructions. The specific primers used for qRT-PCR were designed with Primer Premier 6 software, and their sequences are shown in Table 1. *β-actin* was the internal reference gene. The qRT-PCR reaction system remains consistent with the previous study [18], and its reaction program is as follows: 95℃ for 2 min, 95℃ for 10 s, 59℃ for the 30 s, and 40 cycles of 70℃ for 20 s. The relative expression level of the gene was calculated using the 2^−∆∆CT^ method.

**Table 1:** Primer sequences used for real-time PCR analysis

ID	Gene name	Nucleotide sequence (5’-3’)	Expected product (bp)
CCG027091.1 (HHEX)	Hematopoietically-expressed homeobox protein HHEX	F- GTGGTGCTTCGAGGTTAC R- GTGGCGTGATGTACTTGTA	217
New042111.2 (HSP27)	Heat shock protein 27	F- TGACCTCCGATGACTCTC R- GAACACCTGCGTCTTGAT	179
CCG011464.3 (ERAP1)	Endoplasmic reticulum aminopeptidase 1 isoform X2	F- TTACCTCTCCTCCTATATCACT R- CTCGTCAATTACTATGTCTGTC	223
CCG067658.1 (GSTD1)	glutathione S transferase D1	F- AGACAGACGAGGAGAAGAT R- GGTTGACGACAAGGACTT	240
CCG046938.1 (TGFBI)	Transforming growth factor-beta-induced protein ig-h3	F- TCCACATCATCAACAAGGT R- GGACGAAGAAGGTGTAGAA	183
HM053699.1 (β-actin)	-	F- TCACACACTGTCCCCATCTAG R- ACCACGCTCGGTCAGGATTTTC	114
CCG002672.1 (Rnf14)	Ring Finger Protein 14	F- TGGACTACTTAGACCTCACT R- TCCGTTAGCCACTACACT	218
CCG044698.1 (MRC2)	Mannose Receptor C Type 2	F- CAAGGACGGCAAGTGTTA R- CATACTCGGTCTGGTTGTC	227
CCG018631.1 (GMNN)	geminin	F- AAGGAAGAATGACGAGAACT R- TATGTGGCTGGAGGACTAT	255
CCG013065.1 (TIGD1)	Trigger transposable element–derived 1	F-GTCACTTCATCCTCCTCTG R-AGTTGTCACCATCTTCTTGT	198
EsPVF1 [19]	PDGF/VEGF related factor	F-CCCCGAGGGCTTTGACTA R-TTCCGTTCCTGCTACTGG	152
CCG082508.1 (AKT)	protein kinase B	F- CAACAACGCCTTCTTCCT R- TCCTTCTTCAATCGCTTCAT	267
CCG076881.2 (Caspase-3)	cysteinyl aspartate specific proteinase 3	F- CTATAAGGACCTGACGGAAG R- TGGAGAGGAAGATGTGAGTA	148

### WGCNA analysis

The analysis of gene co-expression module was carried out by WGCNA method and WGCNA software package based on R environment. Firstly, the similarity matrix was generated through computation of the Pearson correlation coefficient between the two gene expressions. Next, a soft threshold β = 19 was chosen to transform the similarity matrix into an adjacency matrix. Then the degree of correlation between genes was characterized by means of the topological overlap (TO), and the adjacency matrix underwent transformation into a topological overlap matrix (TOM). Subsequently, module identification was performed using dynamic tree cut with 1-TOM as the gene clustering distance. Correlations between HIS and modules were calculated to identify significant modules. Finally, the regulatory network was visualized using Cytoscape software.

## Results

### Starvation damages the hepatopancreas of the *E. sinensis*

**Fig. 1:**
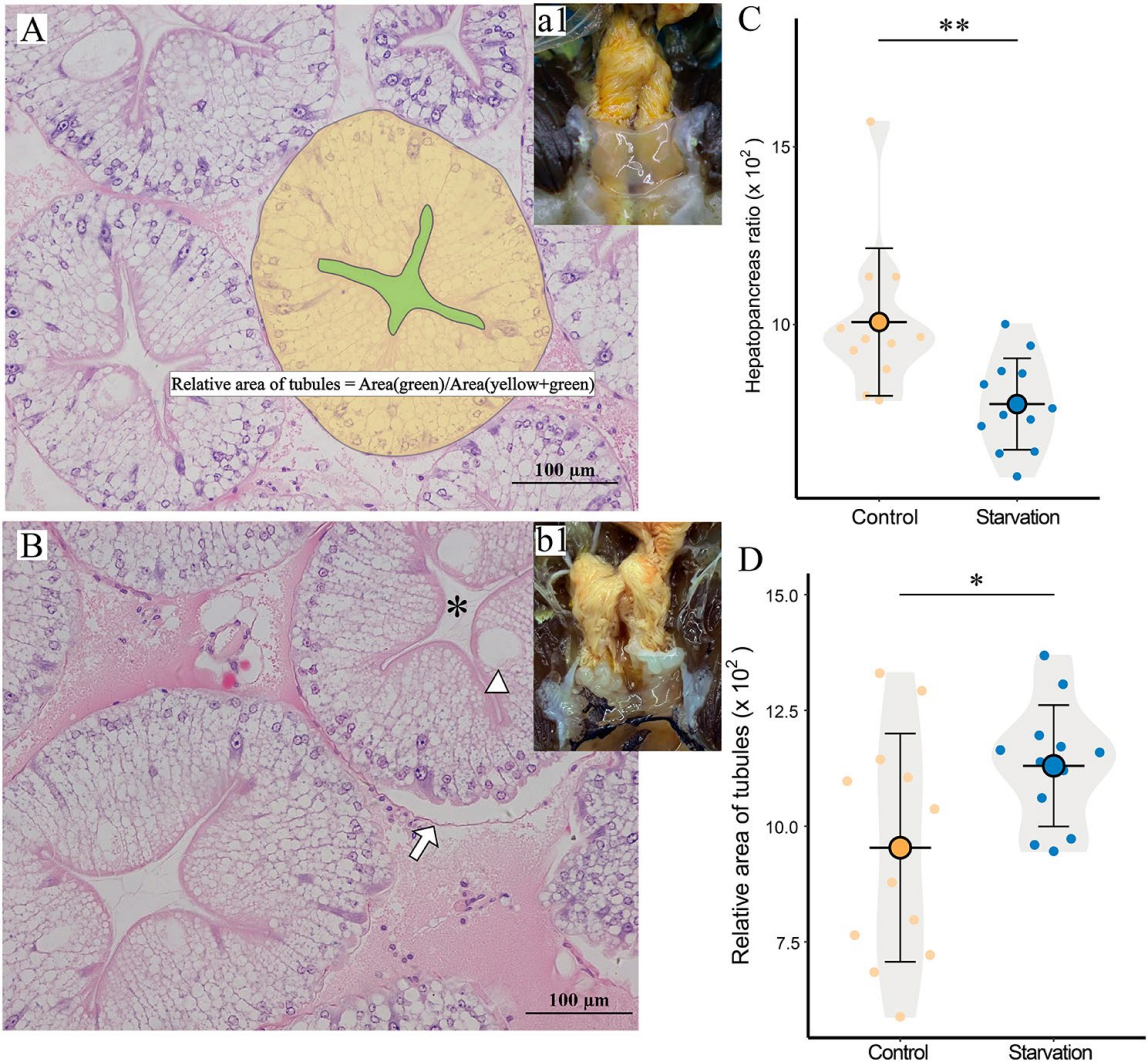
**A**. Hepatopancreas tissue section (H&E) of Chinese Mitten crab in Control group, a1: Clinical symptoms of hepatopancreas in Control group. **B**. Hepatopancreas tissue section (H&E) of Chinese Mitten crab in Starvation goup, b1: Clinical symptoms of hepatopancreas in Starvation group. **C**. Hepatopancreas index of Starvation group and Control group (n = 12). **D**. Relative area of Hepatopancreas tubules between control and starvation groups (n = 12). *: Hepatopancreas tubules; arrowhead: B cell; arrows: basilemma. Data are presented as mean ± standard deviation, *: *P* < 0.05, **:*P* < 0.01

The starvation stress experiment showed that starvation for 21 d did not significantly change the body weight of *E. sinensis* (Fig. 1A). The weight of hepatopancreas was also not significantly changed in the starvation group compared with the control group (Fig. 1B). Anatomical observations revealed that starvation for 21 d resulted in a whitish color of the hepatopancreas (Fig. 1a1, b1). In addition, histopathological observation showed that the basement membrane was separated and the area of the hepatopancreas stellate duct lumen increased in the starved group (Fig. 1A, B, D). By calculating the hepatopancreas index, it was found that starvation caused a significant decrease in the hepatopancreas index of *E. sinensis* (Fig. 1C).

### Transcriptomic analysis

High-throughput sequencing was used to systematically analyze the hepatopancreas genes of *E. sinensis*. 49.4 ± 0.86 and 45.1 ± 0.074 million raw data were obtained from the Control and Starvation groups, respectively. After quality control and contamination removal, high-quality clean data were read, and the clean data were then mapped to the *E. sinensis* reference genome, and the detailed mapping outputs are summarized in Table 2.

**Table 2:** Transcriptome data quality control and comparison table

Sample	Raw reads	Clean reads	Total mapped	Multiple mapped	Uniquely mapped
Control 3	49,019,456	48,033,364	38,865,683(80.91%)	5,437,423(11.32%)	33,428,260(69.59%)
Control 2	50,411,634	48,716,336	39,191,007(80.45%)	3,865,840(7.94%)	35,325,167(72.51%)
Control 1	48,836,520	47,177,704	38,036,864(80.62%)	4,958,658(10.51%)	33,078,206(70.11%)
Starvation 3	45,051,682	43,699,996	35,820,600(81.97%)	4,224,726(9.67%)	31,595,874(72.3%)
Starvation 2	45,201,184	44,022,088	36,162,089(82.15%)	3,662,503(8.32%)	32,499,586(73.83%)
Starvation 1	45,127,488	43,532,614	34,513,189(79.28%)	4,657,270(10.7%)	29,855,919(68.58%)

**Fig. 2:**
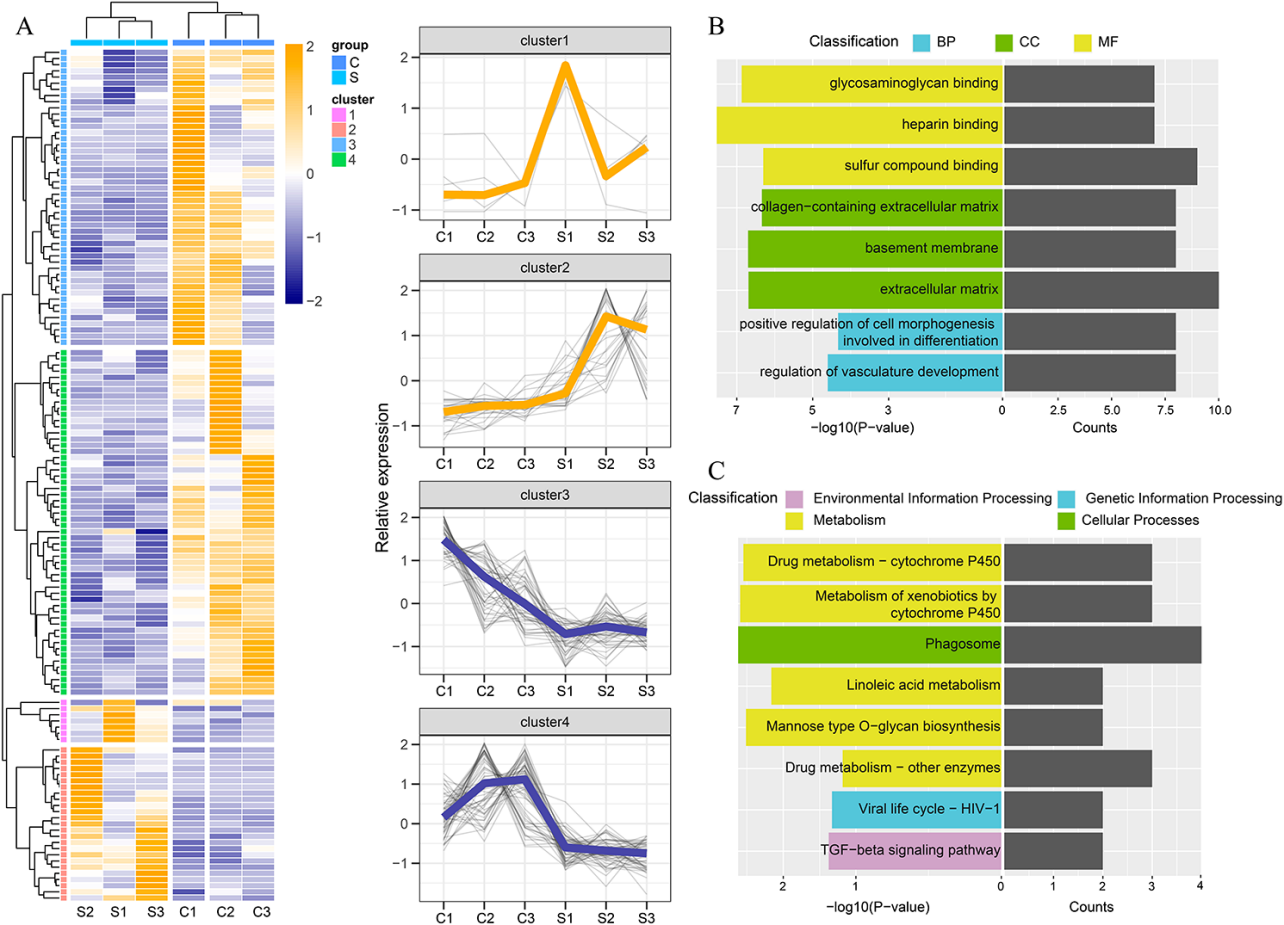
**A**. Hierarchical clustering of differentially expressed genes (DEG) in Starvation group (S) and Control group (C). Normalized expression values (FPKM) for each sample (columns) and gene (rows) are shown in red (up-regulated) and blue (down-regulated) in the heatmap (left side); Expression of genes in each cluster (right side). **B**. Gene Ontology (GO) annotation of significantly different genes. **C**. KEGG annotation of significantly different genes

After 21 days of starvation, the hepatopancreas of *Eriocheir sinensis* exhibited different transcriptome profiles. Principal component analysis (PCA) of the significantly different genes (P < 0.05, fold change > 2) revealed that the groups of samples were clustered together with significant differences (Fig. 2). According to the heat map, there were significant differences in gene expression between the starvation group and the control group. The expression of culster1 and cluster 2 showed mainly an up-regulated state, while the expression of cluster 3 and cluster 4 showed mainly a down-regulated state (Fig. 2A). The GO and KEGG enrichment pathways were sorted according to the number of genes enriched in the pathways and p-value, and the top eight pathways were screened out. GO enrichment analysis revealed that these differential genes were mainly concentrated in the pathways of extracellular matrix, sulfur compound binding, heparin binding, and basement membrane (Fig. 2B). Subsequent KEGG enrichment analysis revealed that five the top eight pathways were classified as Metabolism, while the remaining three were classified as Genetic Information Processing, Environmental information Processing and Cellular Processes, respectively (Fig. 2C).

### Construction of weighted gene co-expression network

**Fig. 3:**
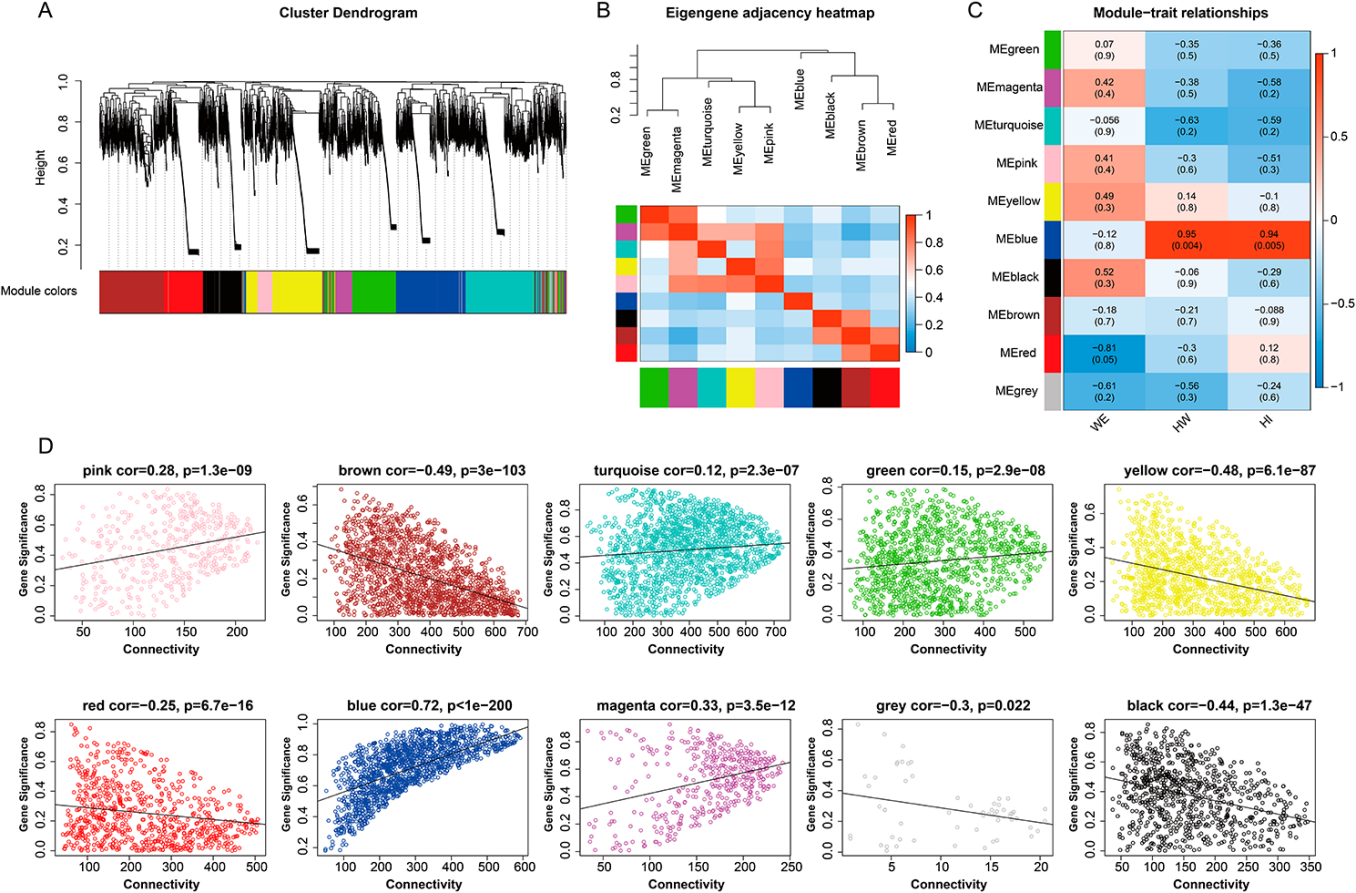
**A**. One-step method for building co-expression networks. **B**. Correlation between modules. **C**. Correlation between modules and body weight (WE), hepatopancreas weight (HW), hepatopancreas index (HI, HI = HW/WE). **D**. Correlations between modules and genes

An analysis of the weighted gene co-expression network (WGCNA) was used to identify co-expressed gene modules and to investigate the relationship between the gene network and the phenotype of interest, as well as the core genes in the network. When power = 19, the fitting index R2 of the scale-free network is 0.8, indicating that the network is close to the scale-free network, which is conducive to subsequent analysis (Fig. 1C). According to the calculated gene dissimilarity, 10 gene co-expression modules were identified (Fig. 3A). Among them, the MEblue module showed a significant positive correlation with the hepatopancreatic index (cor = 0.94, *P* = 0.005) (Fig. 3C), and there was no significant correlation between the MEblue module and other modules (Fig. 3B). To test whether each module gene was closely related to hepatopancreas index, the correlation analysis between hepatopancreas index and each module gene was conducted. The results showed that the correlation coefficient between hepatopanpancreatic index and gene was the highest in MEblue module (cor = 0.72, *P* < 1e-200) (Fig. 3D). That is, the MEblue module has the largest positive correlation with hepatopancreas index, which further confirms the above conclusion. Therefore, this module was selected as an important one for further analysis.

### Genetic screening and qRT-PCR validation

**Fig. 4:**
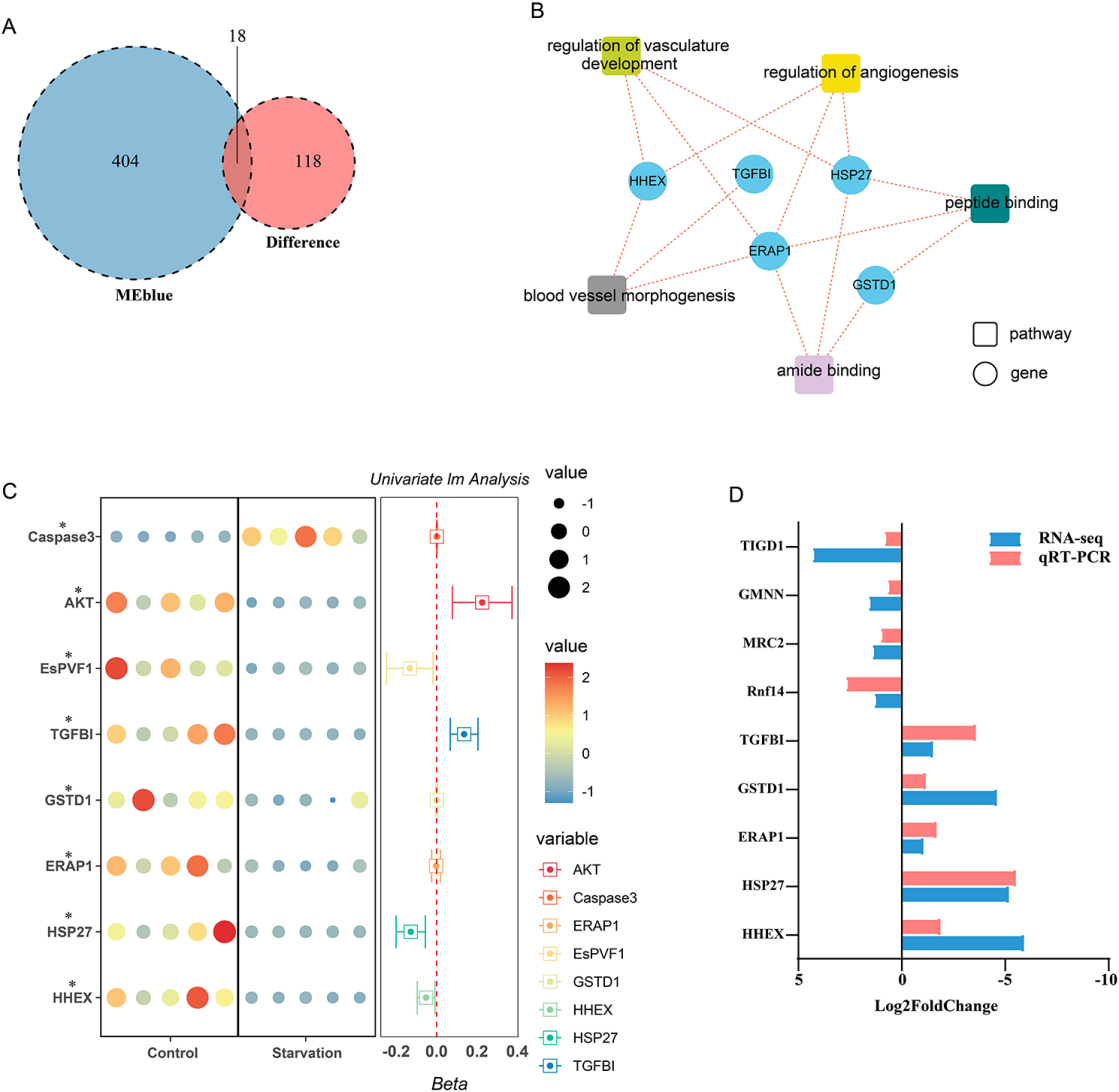
**A**. Venn diagram showing gene distribution between MEblue module and significantly different genes. **B**. GO pathway enrichment analysis of shared genes between MEblue module and significantly different genes. **C**. Linear regression was used to verify the correlation between qRT-PCR results of selected differential genes and hepatopancreas index (HIS) (n = 5). *: *P* < 0.05. D. qRT-PCR validation of the selected genes

Based on the filter (|cor| > 0.9 *P* < 0.05), the genes highly correlated with hepatopancreas index in the MEblue module were screened, and 422 genes were screened out. Subsequently, the 422 genes were analyzed with the differentially expressed genes, and 18 common genes were obtained (Fig. 4A). These 18 genes were used for enrichment analysis, and the top 5 pathways were ranked according to the count and p-value. It was found that these genes mainly focused on regulation of vasculature development, regulation of angiogenesis, peptide binding, blood vessel morphogenesis, and amide binding pathways (Fig. 4B, Table S1_1 and Table S1_2).

Subsequently, the 5 genes (HHEX (CCG027091.1), HSP27 (New042111.2), ERAP1 (CCG011464.3), GSTD1 (CCG067658.1), TGFBI (CCG046938.1)) enriched in the first 5 pathways and 4 genes (Rnf14 (CCG002672.1), MRC2 (CCG044698.1), GMNN (CCG018631.1), TIGD1 (CCG013065.1)) significantly up-regulated were selected for qRT-PCR verification. The results showed that compared with the Control group, the 9 genes in the Starvation group were significantly changed, and the expression patterns were consistent with the transcriptome data, indicating that the transcriptome analysis was credible (Fig. 4D). The expression of AKT, EsPVF1, TGFBI, GSTD1, EARP1, HSP27, and HHEX was significantly lower in the starvation group than in the control group, while the expression of Caspase-3 was the opposite. The prediction results of the linear regression model of hepatopanlet showed that 5 genes (TGFBI, HSP27, HHEX, EsPVF1, AKT) were strongly correlated with HIS, indicating that they were important genes affecting hepatopanlet index (Fig. 4C).

## Discussion

The hepatopancreas of crustaceans is not only the site of digestive enzyme synthesis and secretion, but also the main organ for storing energy, which plays a crucial role in maintaining the life of the body when food is scarce. Previous studies have shown that the HIS of Chinese mitten crab decreased significantly after 21 days of starvation, and with prolonged starvation time till 41 days, hepatopancreas cell apoptosis greatly through the death receptor pathway and mitochondrial pathway [5]. During this process, energy substances such as lipids and glycogen in the hepatopancreatic cells are depleted [5]. As the consumption continues, the organelles of the hepatopancreatic cells are gradually catabolized, resulting in a dramatic change in the area of the hepatopancreatic tubules [5]. Similar results were found in this study, after 21 days of starvation, the hepatopancreas of *E. sinensis* turned slightly white and the HIS decreased significantly as well as the astrocytic lumen was dilated. The expression of Caspase-3 was found to be significantly elevated by the gene assay, which confirms that starvation could further deplete hepatopancreatic cells through apoptosis.

Angiogenesis refers to the process in which blood vessels grow new capillaries on the basis of the original blood vessels [20], which is not only a key process for promoting cell development and wound healing, but also may lead to related diseases. Starvation, an effective signal for angiogenes, directly affects the metabolism of the body. [21]. Studies have found that starvation could inhibit angiogenesis by inducing oxidative stress [22, 23]. In oxidative stress, glutathione plays an important protective role. As the main intracellular antioxidant substance, glutathione has the reducing ability to neutralize and scavenge reactive oxygen and nitrogen species, protecting cells from oxidative damage [24]. Glutamine, a precursor of reduced glutathione (GSH), plays an important role in the regulation of antioxidant responses. Amide binding and peptide binding are two important binding methods involved in glutamine metabolism [25, 26]. In this study, the transcriptome revealed that starvation inhibited amide binding and peptide binding, which led to deranged glutamine metabolism, in turn affecting oxidative regulatory responses, and inhibiting angiogenesis finally.

**Fig. 5:**
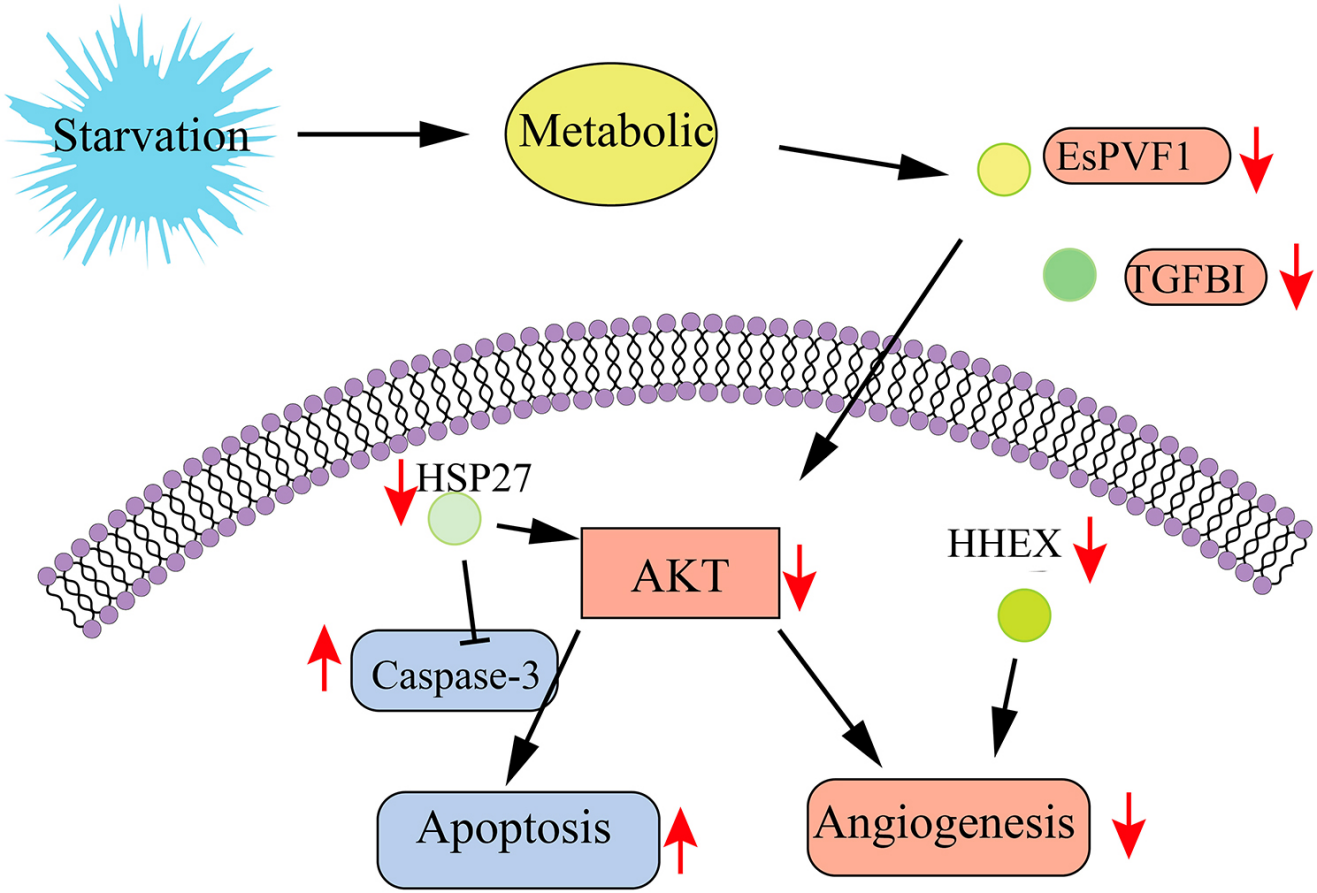
Mechanism of the effect of starvation on hepatopancreas in *Eriocheir sinensis*

EsPVF1 (PDGF/VEGF-related factor from *E. sinensis*) has a similar structure and function to PDGF and VEGF-related factors of other species [19]. PDGF/VEGF-related factors play an important roles in cell signaling and vascular biological processes [19]. All members of the PDGF/VEGF family share the highly conserved and specific PDGF/VEGF homology domains necessary for receptor binding and activation [27]. VGEFA was found to prevent apoptosis induced by serum starvation in vitro [28]. Binding of PDGF and VGEF to their receptors activates downstream signaling molecules, including AKT [29, 30]. Activated AKT can further regulate a variety of cellular processes, including apoptosis and angiogenesis [29, 30]. In addition, several studies found that HHEX, one of the first genes expressed in endothelial cells and hematopoietic precursors in frogs, zebrafish and mice, not only regulates endothelial and hematopoietic differentiation, but also is a transcriptional regulator of the VEGFC/FLT4/PROX1 signaling axis during vascular development and promotes angiogenesis [31]. TGFBI was identified as a TGF-β-inducible gene encoding transforming growth factor β-inducible protein (TGFBIp), a 68 kda extracellular matrix (ECM) protein that plays an important role in various physiological and pathological processes by regulating cell adhesion, proliferation and apoptosis [32]. Previous studies have identified a pro-angiogenic role for TGFBI in CRC cells [33]. Heat shock protein 27 (HSP27), a chaperone protein, not only has the ability to regulate angiogenesis, but also has anti-apoptotic ability [34]. Previous studies have revealed that its anti-apoptotic mechanism is through the binding and inactivation of the pro-apoptotic molecules Smac, caspase 3, caspase 9 and cytochrome c, and activation of AKT [34, 35]. Caspase-3, a member of the caspases family, is considered to perform apoptosis [36]. AKT is a serine/threonine kinase that regulates multiple cellular processes, promotes cell survival and inhibits apoptosis [36]. The balance between these two proteins and their activities is essential for maintaining cellular homeostasis [34, 36]. In this study, it was found that starvation caused down-regulation of TGFBI, HSP27, HHEX, EsPVF1 and AKT genes in the hepatopancreas of *E. sinensis* and promoted the expression of Caspase-3. Among them, TGFBI, HSP27, HHEX, EsPVF1 and AKT genes were strongly correlated with hepatopancreas index. It was hypothesized that starvation may regulate AKT through TGFBI, HSP27, HHEX and EsPVF1 to inhibit angiogenesis and promote apoptosis (Fig. 5).

## Conclusion

In this study, we found that the hepatopancreas index of *E. sinensis* decreased significantly after 21 days of starvation, and the hepatopancreas showed signs of atrophy. Further transcriptome analysis revealed that this phenomenon was accompanied by the inhibition of angiogenesis and metabolic abnormalities. Based on gene expression, it is speculated that starvation inhibits AKT signaling by decreasing the expression of TGFBI, HSP27, HHEX, and EsPVF1, thereby hindering angiogenesis and promoting apoptosis, leading to hepatopancreas atrophy.

### Electronic supplementary material

Below is the link to the electronic supplementary material.

Supplementary Material 1

Supplementary Material 2

## Data Availability

The datasets supporting the conclusions of this article are included within the article. The data used for transcriptome analysis could obtained in the Genebank (NCBI: PRJNA918439, https://www.ncbi.nlm.nih.gov/bioproject/PRJNA918439).
