# Supplementary material for: Starvation induces hepatopancreas atrophy in Chinese mitten crab (Eriocheir sinensis) by inhibiting angiogenesis
Source: BMC Genomics. 2023 Oct 12;24:612. doi: 10.1186/s12864-023-09620-x (PMC10571328; doi:10.1186/s12864-023-09620-x)
Supplement: Supplementary file 2 — Supplementary Material 2 [file 12864_2023_9620_MOESM2_ESM.docx]

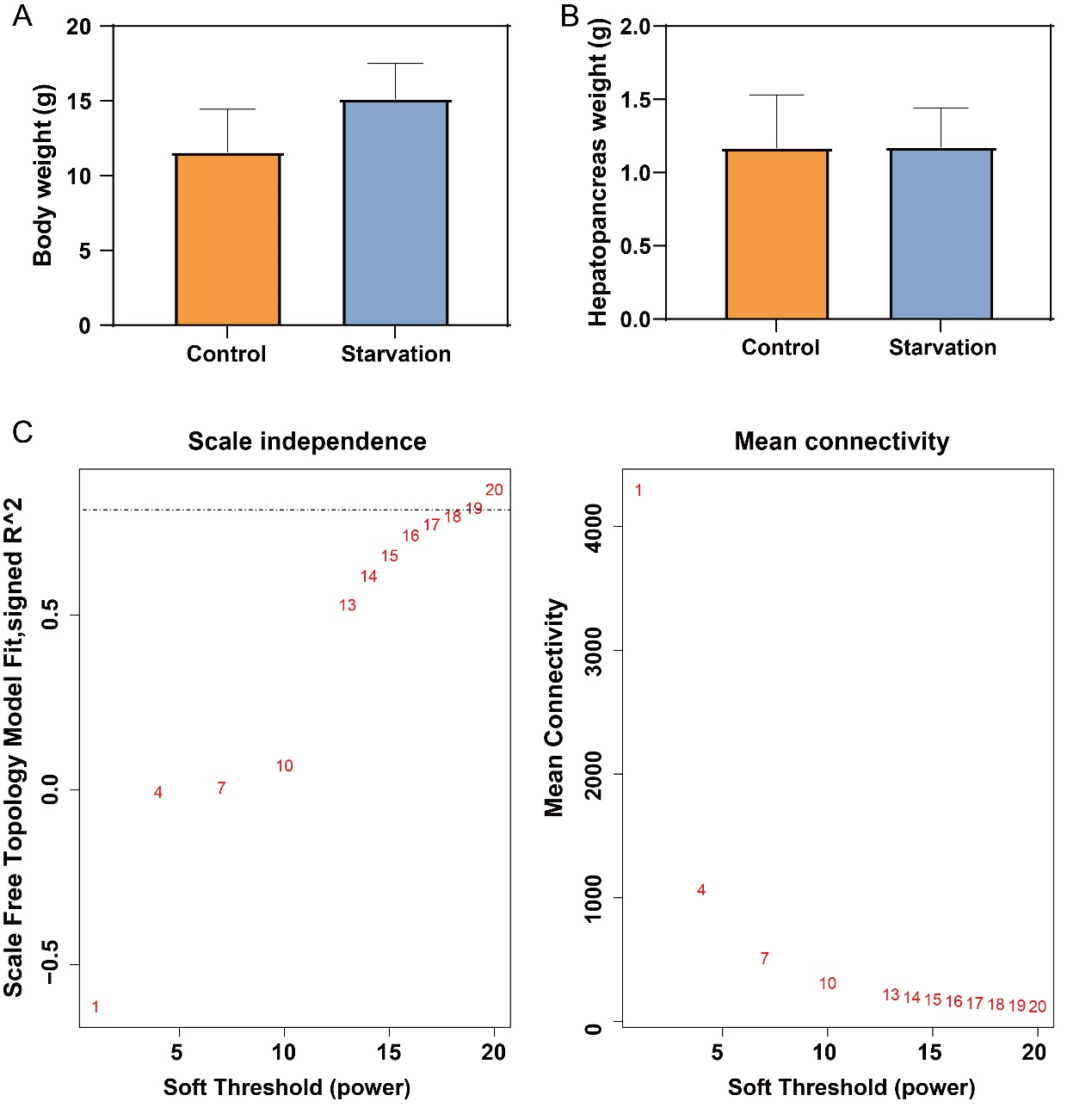


FigS1 A. Body weight of Starvation group and Control group. B. Hepatopancreas weight of Star-vation group and Control group. C. Analysis of the scale-free index for various soft-threshold powers (β). Data are present-ed as mean ± standard deviation, *: P<0.05.


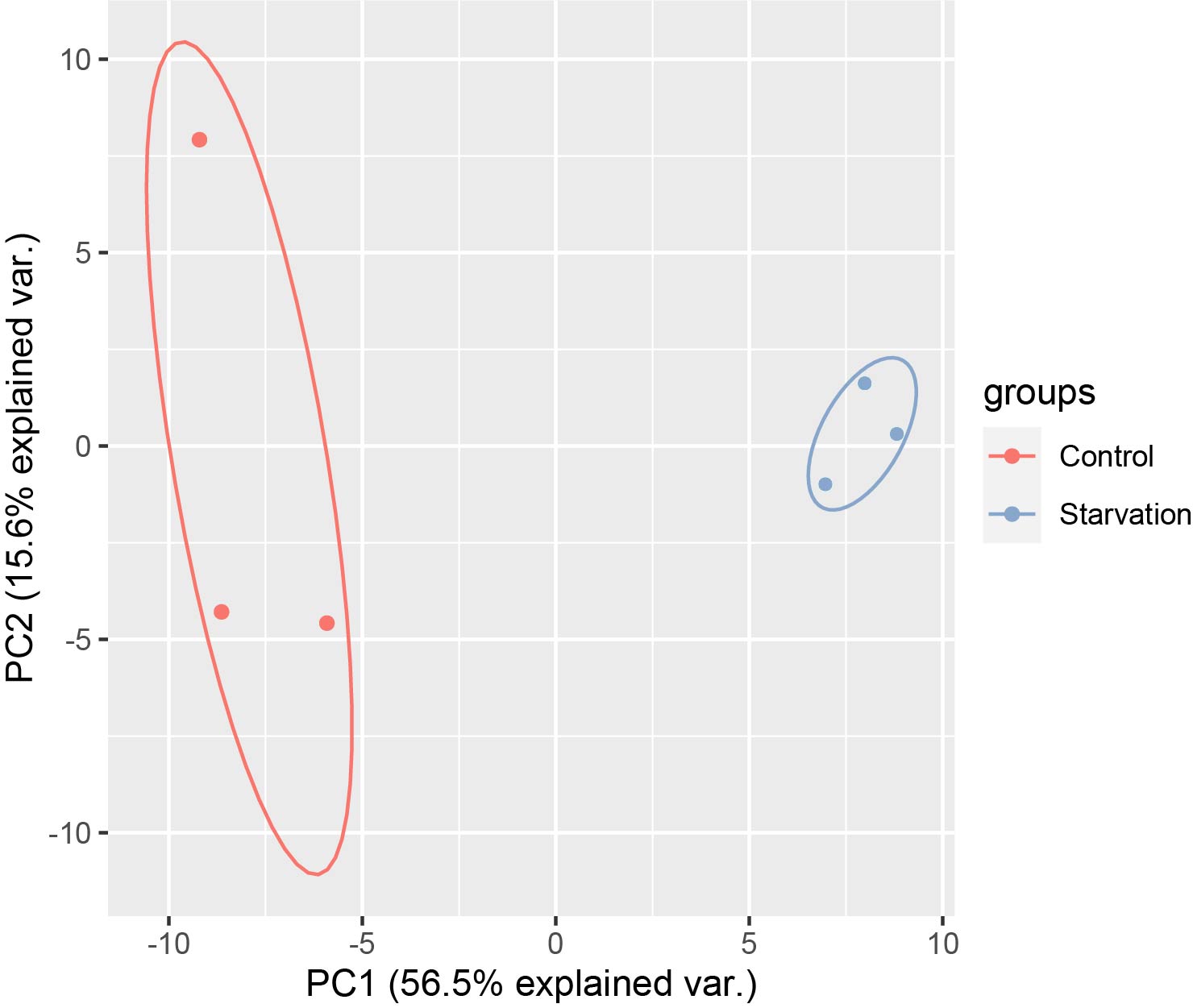


FigS2. Principal component analysis of transcriptome data from hepatopancreas tissues of Eriocheir sinensis in control and starvation groups. The horizontal and vertical coordinates indicate the first and second principal components respectively, and the contribution of each principal component is shown in parentheses.
